# Lecture on the Diseases of the Urinary Organs

**Published:** 1843-02-18

**Authors:** 


### BIBLIOGRAPHICAL NOTICES.

*Lectures on the Diseases of the Urinary Organs.* By Sir Benjamin C. Brodie, Bart. F. R. S. Serjeant Surgeon to the Queen. From the third London edition, with alterations and additions. Philadelphia: 1843. 8vo. pp. 214.

Sir Benjamin C. Brodie is confessedly at the head of British surgery. He is known for his sound judgment and the practical character of his mind. These qualities, joined to an unrivalled experience in the metropolis of the civilized world for some thirty years, are calculated to excite high expectations of excellence in any professional production emanating from him. The publication of the present work will not disappoint these hopes. In it Sir B. Brodie fully sustains his reputation. The work is the result of the author's abundant experience; and the excellent curative recommendations, modestly inculcated, come from one perfectly familiar with the subject. This is a signal excellence over the crude compilations we are so constantly called on to notice. Its appearance is opportune, supplying as it does, in a measure, a great want in English medical literature. It is by no means a complete treatise on the diseases of the genito-urinary apparatus, but those affections which are treated are ably and fully handled.

The work is a valuable contribution to the department of surgery of which it treats. We strongly and cordially recommend it.

The first part of the volume is dedicated to the diseases of the male urethra—stricture, and its consequences, urinary abscesses and fistulæ. Too little perhaps is said of the pathology of this common, but ill understood affection; and the treatment by the nitrate of silver is rather hastily dismissed, when its importance in certain cases is considered. In speaking of Dr. Arnott's new method,—the introduction of a tube of varnished silk into the stricture, and its dilatation by the impulsion of air by a syringe—Sir B. Brodie objects to the practice as one not calculated to fulfil the proposed intentions. The great danger, we conceive, of Dr. Arnott's plan, is the risk of rupture of the urethra, especially when the unsupported membranous portion is the seat of disease. A resort to cutting instruments is unequivocally condemned.

Several good chapters on some of the diseases of the bladder and of the prostate gland next follow. The last six chapters are on calculus vesicæ and its treatment. This is the most interesting part of the work, and we regret not being able to enter more into details upon it. This subject is fully and ably handled, and we especially commend these lectures to the study of the reader. They will amply reward the time and labour bestowed on them. The concluding chapter is on Lithotripsy. In the former edition of this work, Sir B. Brodie did not feel himself "competent to offer more than a few general observations. I have now ventured to discuss this new method of treatment more at length, giving some practical instructions for the performance of the operation, which may probably be acceptable to the younger members of our profession, and to those whose minds have not yet been directed to the subject, at the same time endeavouring to assign to it what I believe to be its proper place among the appliances of surgery, and what, if I am not greatly mistaken, will be conceded to it by others, when time and experience shall have dissipated alike the prejudices of those who underrate its importance and usefulness, and of those who hold it to be more really useful than it really is."

